# Clinical significance of Philadelphia‐like‐related genes in a resource‐constrained setting of adult B‐acute lymphoblastic leukemia patients

**DOI:** 10.1002/jha2.1030

**Published:** 2024-10-07

**Authors:** Keli Lima, César Alexander Ortiz Rojas, Frederico Lisboa Nogueira, Wellington Fernandes da Silva, Rita de Cássia Cavaglieri, Luciana Nardinelli, Aline de Medeiros Leal, Elvira Deolinda Rodrigues Pereira Velloso, Israel Bendit, Alvaro Alencar, Charles G. Mullighan, João Agostinho Machado‐Neto, Eduardo Magalhães Rego

**Affiliations:** ^1^ Department of Internal Medicine, Hematology Division, Laboratory of Medical Investigation in Pathogenesis and Targeted Therapy in Onco‐Immuno‐Hematology (LIM‐31), Faculdade de Medicina University of São Paulo Sao Paulo Brazil; ^2^ Department of Pharmacology, Institute of Biomedical Sciences University of São Paulo Sao Paulo Brazil; ^3^ Sylvester Comprehensive Cancer Center University of Miami Miller School of Medicine Miami Florida USA; ^4^ Department of Pathology St. Jude Children's Research Hospital Memphis Tennessee USA; ^5^ Center of Excellence for Leukemia Studies St. Jude Children's Research Hospital Memphis Tennessee USA

Dear Editor,

The t(9;22)(q34;q11) translocation, which produces *BCR::ABL1* (Ph^+^), a constitutively active tyrosine kinase, occurs in approximately 25% of adult patients with acute lymphoblastic leukemia (ALL). Before the advent of tyrosine kinase inhibitors (TKIs), this molecular subtype was associated with unfavorable clinical outcomes [1, 2]. The incorporation of TKIs resulted in significant improvements in overall survival (OS), event‐free survival (EFS), and rates of complete remission (CR). For example, the addition of the TKI dasatinib to chemotherapy resulted in higher rates of complete molecular response (CMR) and lower rates of relapse compared to chemotherapy alone [3].

In 2016, the World Health Organization recognized a new provisional diagnostic entity called “Philadelphia‐like” (Ph‐like) or “*BCR::ABL1*‐like” ALL, which refers to a subtype of the B‐ALL precursor that, despite presenting a gene signature and molecularly similar to Ph^+^ ALL, does not present the BCR::ABL1 fusion protein [4, 5, 6]. This group of patients exhibits poor clinical outcomes and presents multiple rearrangements, mutations, and copy number variations involving kinase or cytokine receptor genes, which lead to the activation of JAK2/STAT, ABL1, and RAS signaling pathways [7]. Although several clinical trials have investigated the efficacy of JAK‐ or ABL‐directed TKIs in Ph‐like ALL, the standard of care is still to be determined.

The identification of patients with Ph‐like ALL remains challenging in clinical practice, as methodologies that comprehensively evaluate gene expression are required. In this regard, Chiaretti et al. [8] proposed a tool based on the expression of 10 genes by quantitative polymerase chain reaction (qPCR) and a statistical model (10‐gene score) for screening patients with Ph‐like ALL.

In the present study, we investigated the expression of Ph‐like‐related genes [8] in samples from healthy donors (*n* = 12) and adult patients with B‐ALL (*n* = 83 [Ph^+^ *n* = 33 and Ph^−^ *n* = 50]) and their association with clinical and laboratory characteristics and survival outcomes. The research protocol was approved by the Committee of Ethics in Hospital das Clínicas of the Faculty of Medicine of the University of São Paulo (CAAE: 32409120.0.0000.0068). An overview of patient characteristics is provided in Table S1. Briefly, those patients were treated according to their Philadelphia status and age, regimens were described elsewhere [9]. Total RNA was extracted from bone marrow mononuclear cell samples, with subsequent complementary DNA (cDNA) synthesis from 1 µg of RNA accomplished using the High‐Capacity cDNA Reverse Transcription Kit (Thermo Fisher Scientific). qPCR analysis was executed on a QuantStudio 3 Real‐Time PCR System, employing a SybrGreen System (Thermo Fisher Scientific) to evaluate the expression levels of Ph‐like‐related genes (Table S2). The reference genes used for normalization were *ACTB* and *GAPDH*. Relative quantification values were determined using the 2^−ΔΔCT^ equation [10]. The statistical model proposed by Chiaretti et al. [8] to obtain scores was applied. For the 10‐gene score and individual gene expression, dichotomization was carried out utilizing the receiver operating characteristic curve, incorporating metrics such as the area under the curve (AUC) and the Youden index. The process involved a maximization metric facilitated by the R package cutpoint [11]. Comparisons between groups were performed using the Mann‐Whitney test, Fisher's exact test, or chi‐square test, as appropriate. Survival analyses were performed by Kaplan Meier method and Cox regression analysis. A *p* < 0.05 was considered statistically significant.

Gene expression of *CD97*, *CD99*, *CRLF2*, *IFITM1*, *IFITM2*, *NUDT4*, *SEMA6A*, *SOCS2*, and *TP53INP1* was higher in B‐ALL patients compared to healthy donors (all *p* < 0.05; Figure 1). In addition, the 10‐genes score was higher in patients with Ph^+^ B‐ALL than in Ph^−^ B‐ALL (*p* < 0.0001; Figure S1). In our cohort, Ph^+^ or Ph^−^ status did not predict the prognosis of patients with B‐ALL (Figure 2A), but higher values in the 10‐genes score were associated with reduced overall survival (Figure 2B). Among Ph^−^ B‐ALL patients, a high score was also associated with lower overall survival rates (*p* = 0.04, Figure 2C). When Ph‐like‐related genes were evaluated individually, only high *IGJ* expression was associated with worse clinical outcomes (Figure S2). In the whole B‐ALL cohort, among the clinical‐laboratory characteristics, higher values in the 10‐genes score were associated with lower LDH levels (*p* < 0.05; Table S1). In the subgroup of Ph^−^ B‐ALL patients, higher values in the score were associated with fewer blasts infiltrating the bone marrow and lower LDH levels (*p* < 0.05; Table S3). In ALL patients, LDH levels were positively correlated with WBC (*r* = 0.32, *p* = 0.0048) and negatively correlated with the 10‐genes score (*r* = ‐0.24, *p* = 0.03), but the 10‐genes score did not correlate with WBC (*r* = 0.02, *p *= 0.88; Figure S3), suggesting that the 10‐genes score may be more associated with resistance to therapy than with a disease with high tumor burden or proliferative phenotype, which requires future investigations. Of note, in both the whole B‐ALL cohort and the Ph^−^ B‐ALL subgroup, the 10‐gene score was an independent prognostic factor in multivariate Cox regression analysis with the covariates gender, age, hematimetric parameters, LDH, and cytogenetic risk (*p* < 0.05, Tables S4 and S5).

**FIGURE 1:**
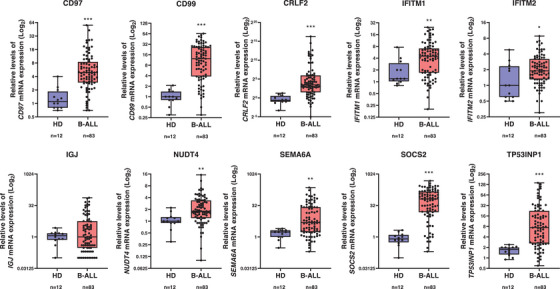
**Genes associated with the Ph‐like signature are highly expressed in adult patients with B‐acute lymphoblastic leukemia (B‐ALL)**. The messenger RNA (mRNA) levels of 10 genes previously associated with Ph‐like (*CD97, CD99, CRFL2, IFITM1, IFITM2, IGJ, NUDT4, SEMA6A, SOCS2*, and *TP53INP1*) were compared between samples from healthy donors (n = 12) and patients with B‐ALL (*n* = 83) by quantitative PCR. *ACTB* and *GAPDH* were used as reference genes. A normal bone marrow sample was used as a calibrator sample. * *p* < 0.05, *** *p* < 0.01, *** *p* < 0.0001; Mann‐Whitney test.

**FIGURE 2:**
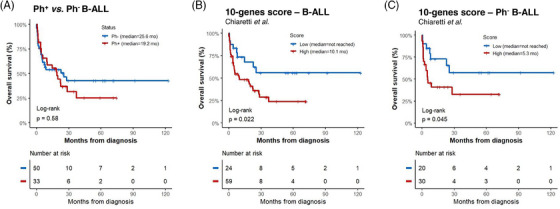
**High scores based on genes associated with Ph‐like predict unfavorable overall survival in patients with B‐acute lymphoblastic leukemia (B‐ALL)**. Kaplan‐Meier curves represent overall survival for patients with B‐ALL dichotomized according to Ph^+^ and Ph^−^ status (A) and according to the 10‐genes score in the whole cohort (B) or in the Ph^−^ cohort (C). The *p*‐values and number of patients at risk are indicated in the log‐rank test.

In the study by Chiaretti et al. [8], both adult and pediatric patients with Ph^−^ B‐ALL were included. This contrasts with our present study, where only adult patients with B‐ALL (Ph^+^ and Ph^−^) were included. An additional noteworthy aspect of our study is that, through unsupervised analysis, the score derived from these 10 genes demonstrated superior efficacy in identifying a risk group compared to the stratification into Ph^+^ or Ph^−^.

Recognizing the significance of identifying the Ph‐like signature for the classification and risk stratification of patients with B‐ALL, some research groups have proposed alternative combinations of genes. For instance, Gupta et al. [12, 13] put forward a set of 8 genes (*JCHAIN*, *CA6*, *MUC4*, *SPATS2L*, *BMPR1B*, *CRLF2*, *ADGRF1*, and *NRXN3*) for this purpose. Notably, the *JCHAIN* (also known as *IGJ*) and *CRLF2* genes overlap with the gene set proposed by Chiaretti et al. [8]. In a recent report, Gestrich et al. [14] highlight that the expression of IGJ and SPATS2L, as assessed by immunohistochemistry, sensitively and specifically identifies Ph^+^ and Ph‐like B‐ALL.

Another interesting finding was the observation that only half of the *CRLF2*‐rearranged cases (detected by fluorescence in situ hybridization) had a high 10‐gene score. This underscores the fact that a substantial proportion of these cases may not exhibit a Ph‐like signature. In fact, in the original report by Harvey et al. [15], approximately 37% of *CRLF2*‐rearranged cases did not display a Ph‐like signature according to gene expression profiling. This is significant, as in many settings where a comprehensive gene expression approach is not available, *CRLF2* rearrangement has been treated as synonymous with Ph‐like disease.

Our study paves the way for the application of a risk score for adult patients with B‐ALL based on gene expression that is easy to perform in centers with limited resources; however, it has limitations. In the B‐ALL cohort studied, there is no large‐scale gene expression assessment data (i.e., RNA‐seq or cDNA microarray), which does not allow us to classify the patients included as Ph‐like. Furthermore, our cohort comes from a single center, requiring validation by other research groups in future studies.

In summary, the differential expression of the evaluated genes deserves further investigation as it may have implications for the biology of the disease and become therapeutic targets. The score based on Ph‐like genes may be a useful tool for risk stratification of adult patients with B‐ALL in settings with limited resources.

## AUTHOR CONTRIBUTIONS

*Conceptualization*: Keli Lima, João Agostinho Machado‐Neto, and Eduardo Magalhães Rego. *Investigation*: Keli Lima, César Alexander Ortiz Rojas, Frederico Lisboa Nogueira, Wellington Fernandes da Silva, João Agostinho Machado‐Neto, and Eduardo Magalhães Rego. *Technical assistance and discussion*: Rita de Cássia Cavaglieri, Luciana Nardinelli, Aline de Medeiros Leal, Elvira Deolinda Rodrigues Pereira Velloso, Israel Bendit, Alvaro Alencar, and Charles G. Mullighan. *Resources*: João Agostinho Machado‐Neto and Eduardo Magalhães Rego. *Data curation*: Keli Lima, César Alexander Ortiz Rojas, Frederico Lisboa Nogueira, Wellington Fernandes da Silva, João Agostinho Machado‐Neto, and Eduardo Magalhães Rego. *Provision of study patients*: Wellington Fernandes da Silva, Elvira Deolinda Rodrigues Pereira Velloso, and Israel Bendit. *Writing—review and editing*: All authors. *Funding acquisition*: João Agostinho Machado‐Neto, and Eduardo Magalhães Rego. *Overall supervision*: Eduardo Magalhães Rego.

## CONFLICT OF INTEREST STATEMENT

The authors declare no conflict of interest.

## FUNDING INFORMATION

This work was supported by the São Paulo Research Foundation (FAPESP) [grant numbers 2021/11606‐3 and 2020/12842‐0]. This study was also financed in part by the Coordenação de Aperfeiçoamento de Pessoal de Nível Superior‐Brasil (CAPES)‐Finance Code 001.

## ETHICS STATEMENT

All procedures performed in this study involving human participants were in accordance with the ethical standards of the institutional research committee and with the 1964 Helsinki Declaration and its later amendments or comparable ethical standards.

## PATIENT CONSENT STATEMENT

The authors have confirmed patient consent statement is not needed for this submission

## CLINICAL TRIAL REGISTRATION

The authors have confirmed clinical trial registration is not needed for this submission.

## Supporting information



Supporting Information

Supporting Information

Supporting Information

Supporting Information

Supporting Information

Supporting Information

Supporting Information

Supporting Information

## Data Availability

Data are available upon request from the authors.
