# Supplementary figures and images for: Clinical significance of Philadelphia‐like‐related genes in a resource‐constrained setting of adult B‐acute lymphoblastic leukemia patients
Source: EJHaem. 2024 Oct 7;5(6):1366–9. doi: 10.1002/jha2.1030 (PMC11647732; doi:10.1002/jha2.1030)

# B-ALL

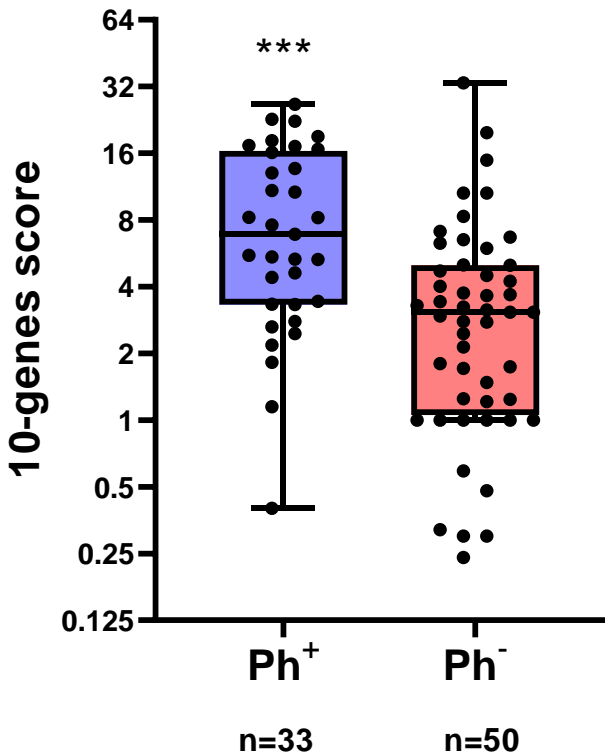

Supplement: Supplementary file 1 — Supporting Information [file JHA2-5-1366-s001.pdf]

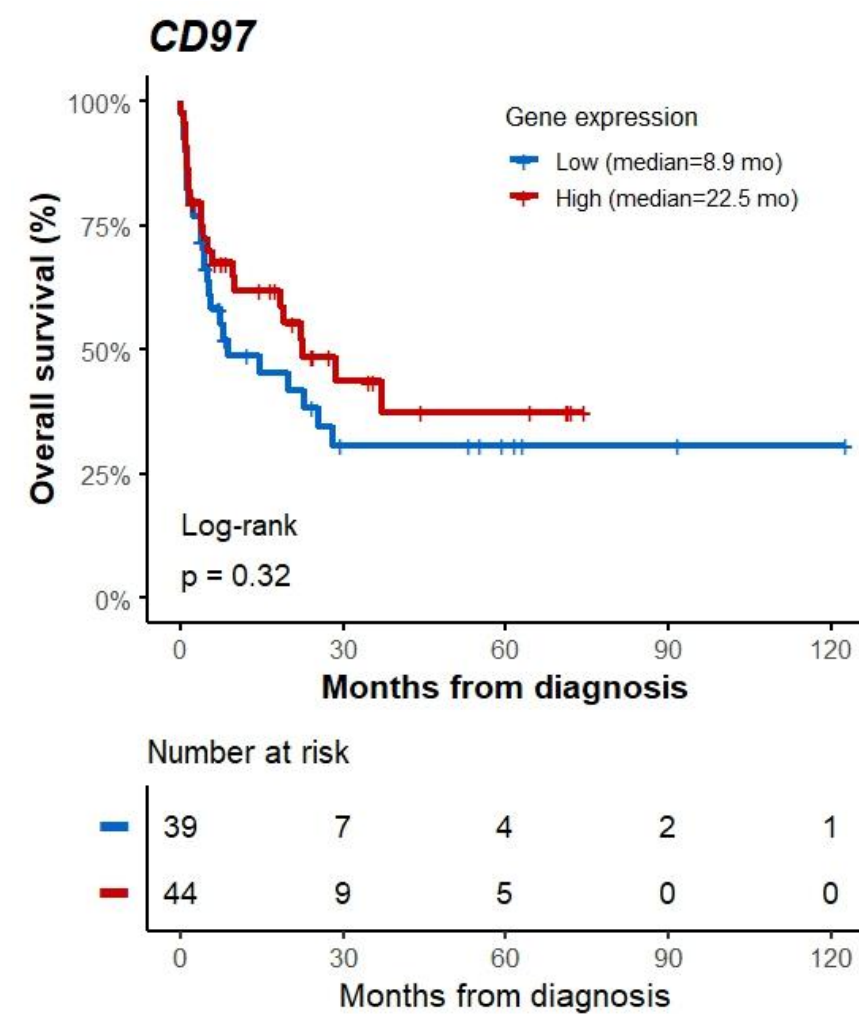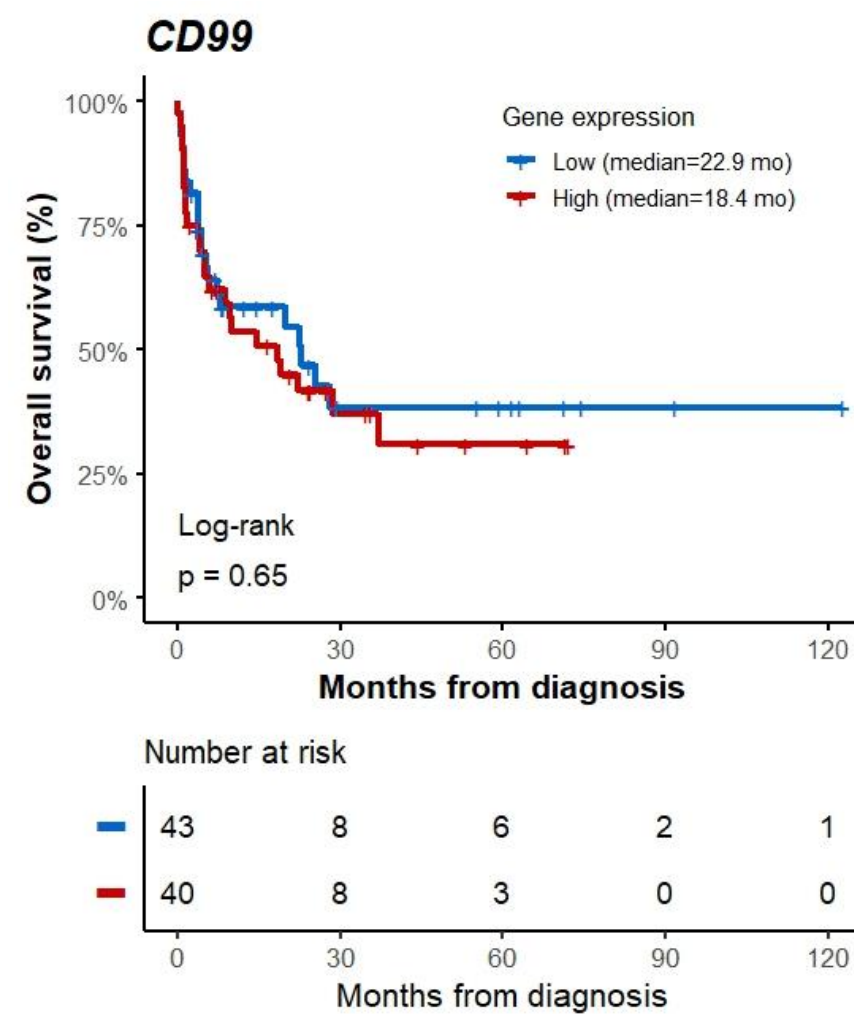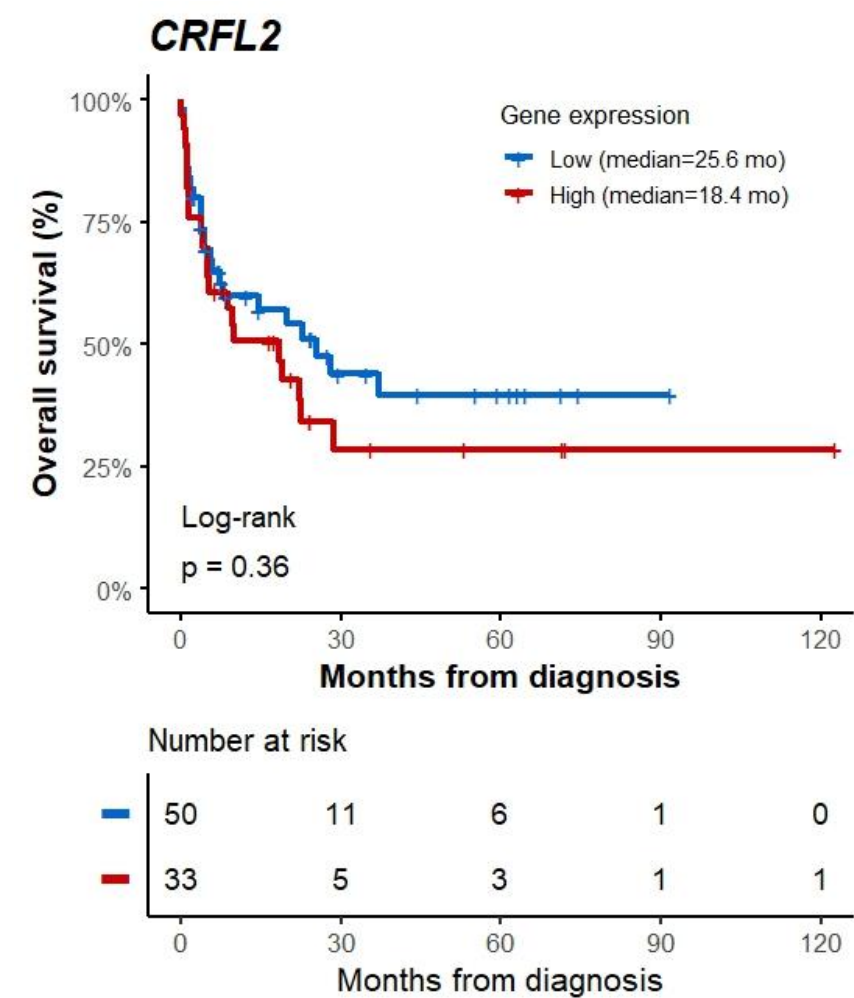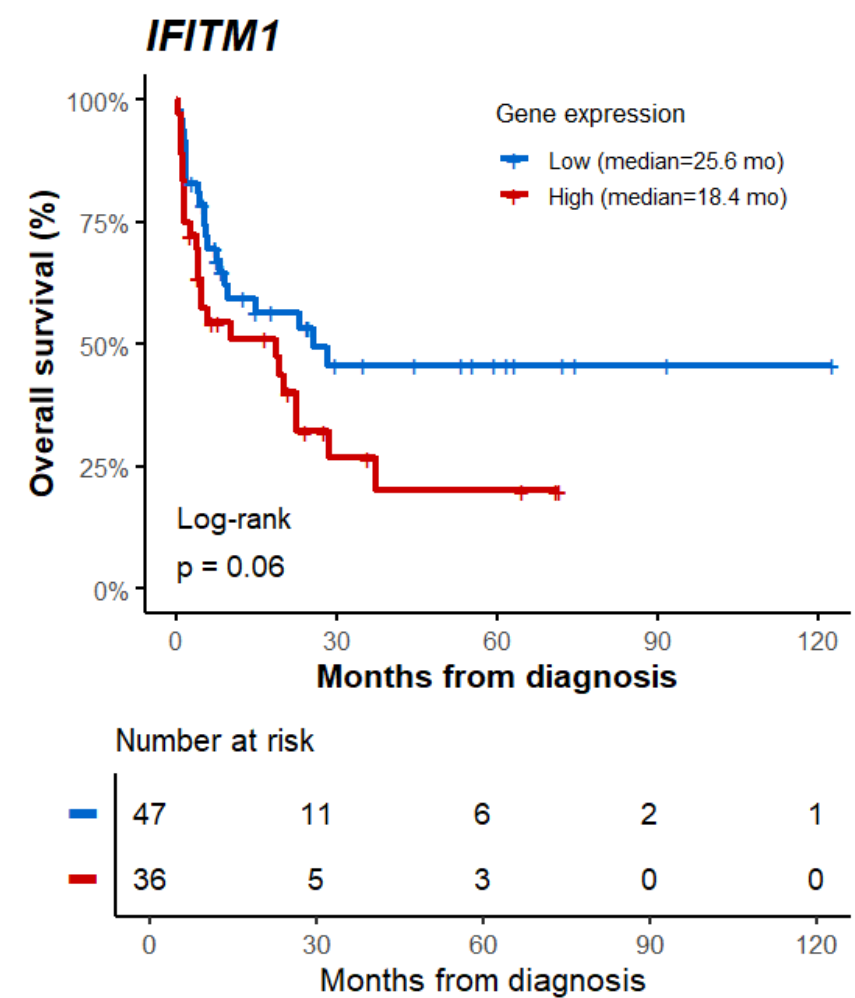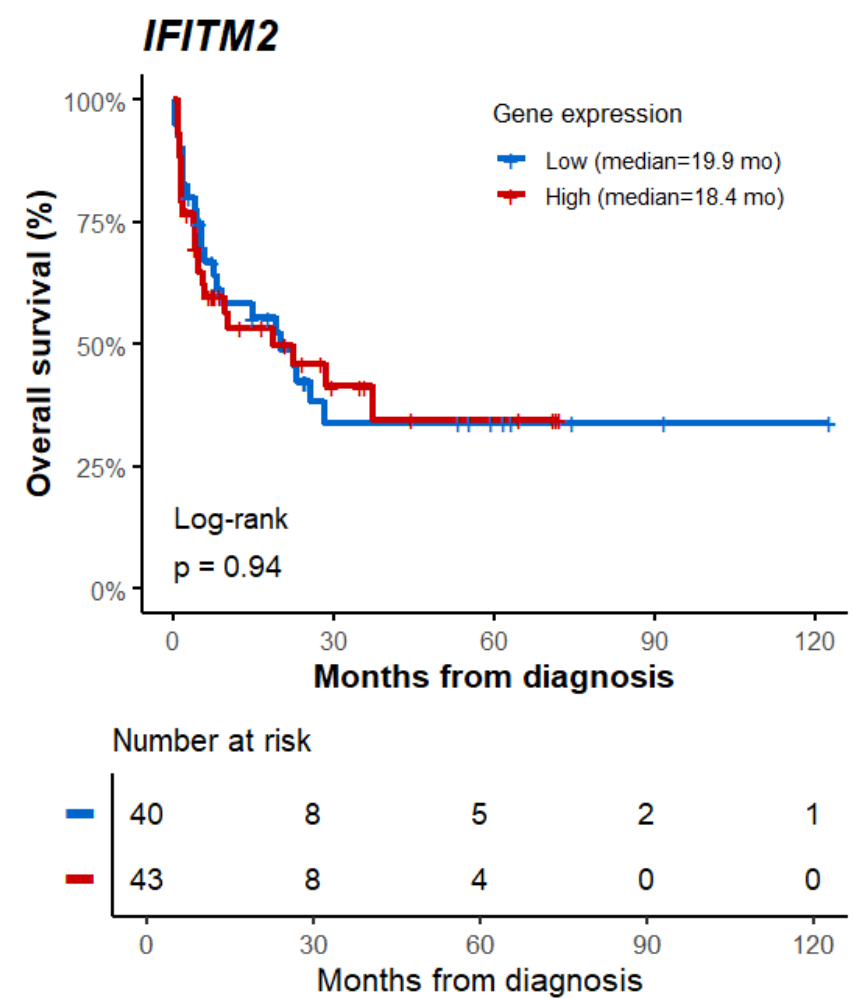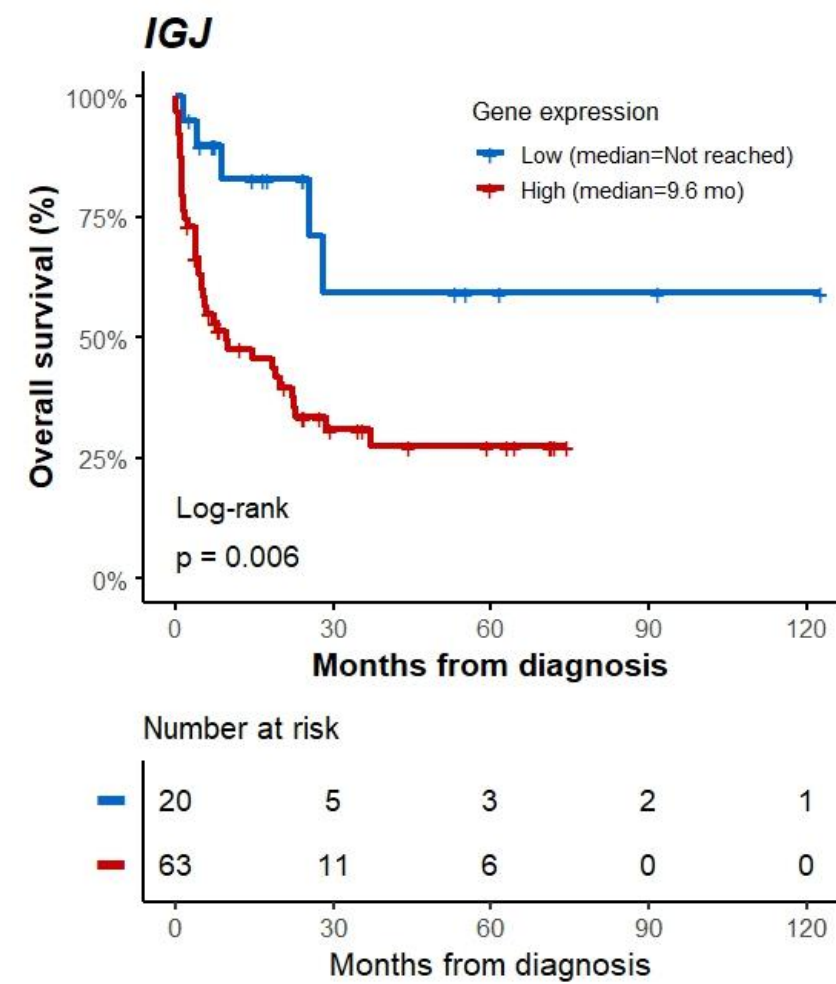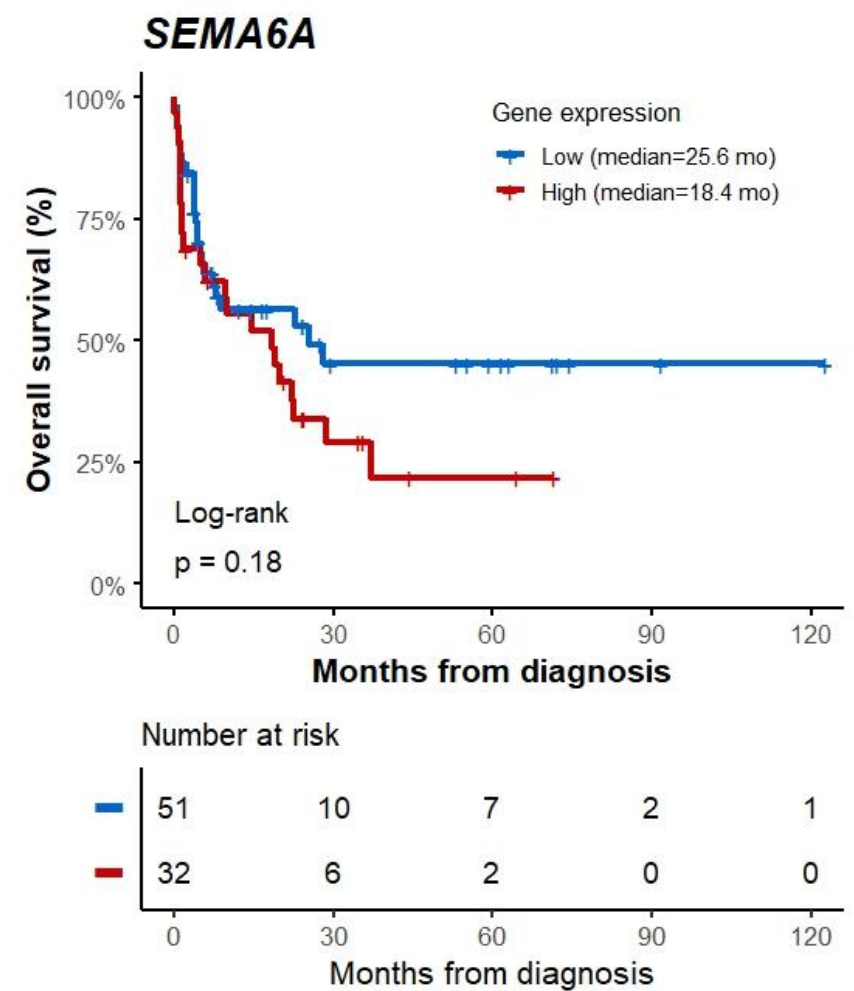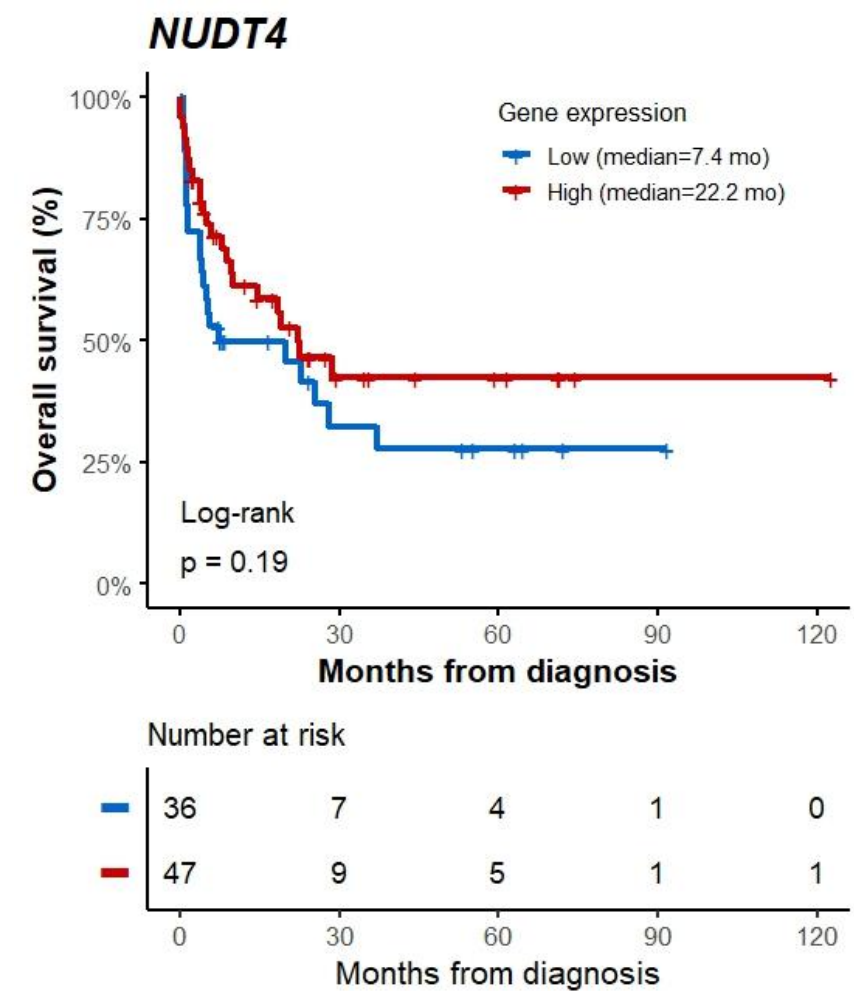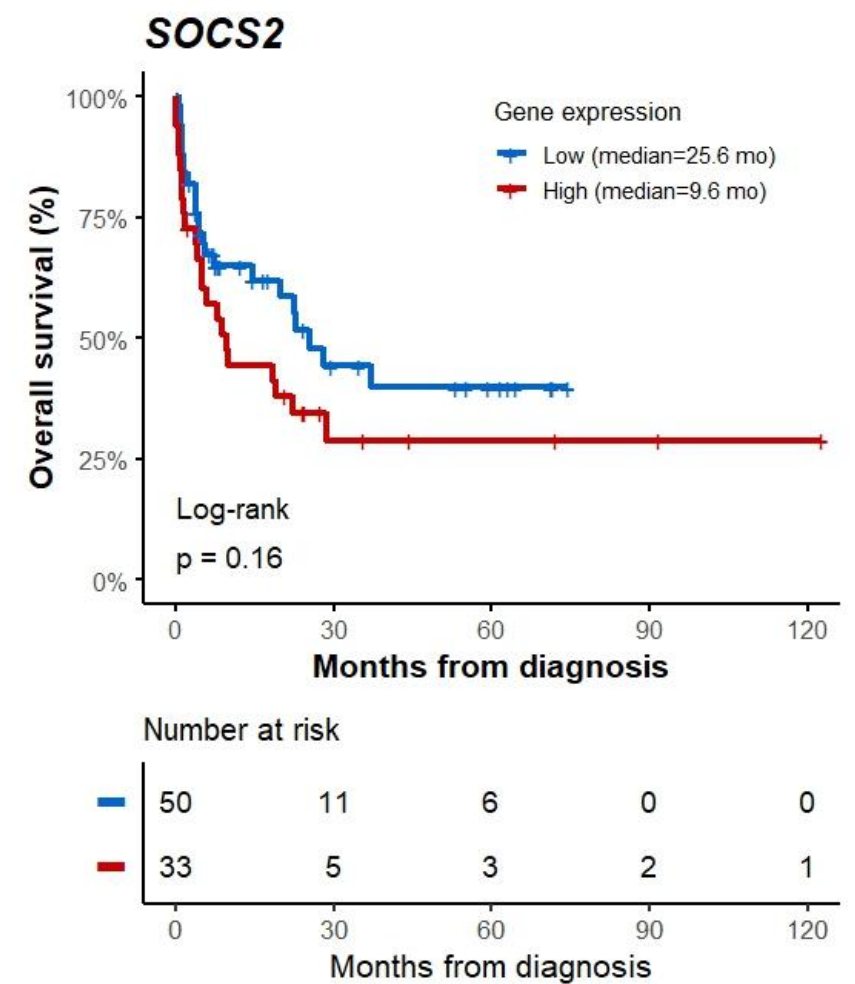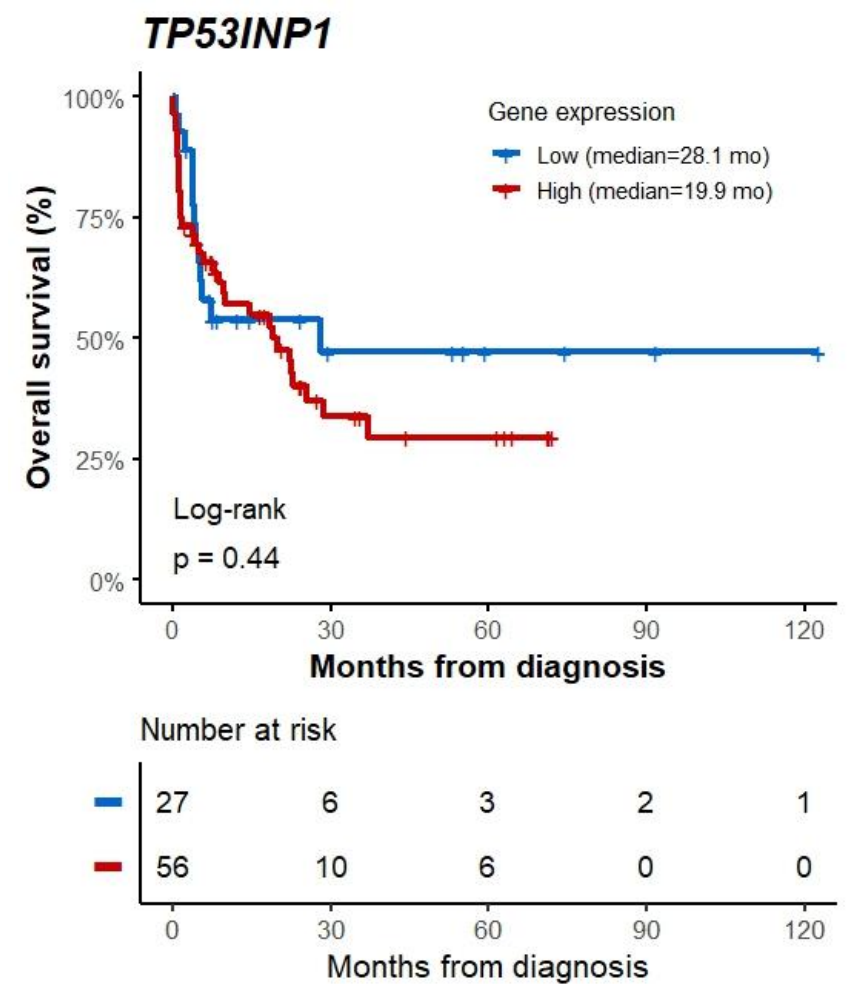

Supplement: Supplementary file 2 — Supporting Information [file JHA2-5-1366-s002.pdf]

ALL patients

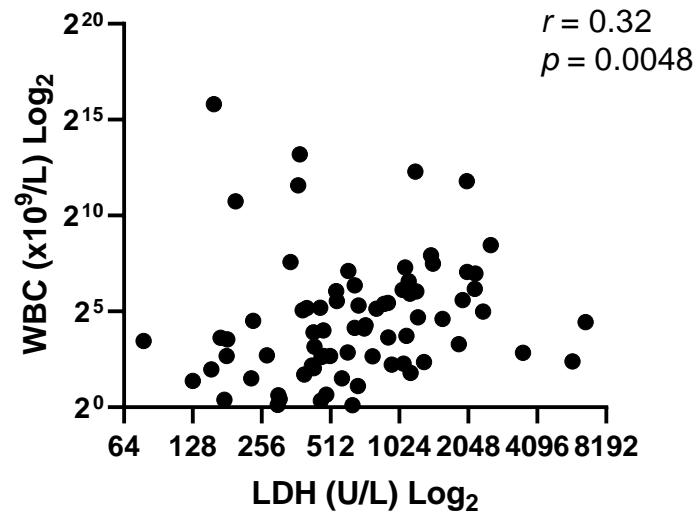

ALL patients

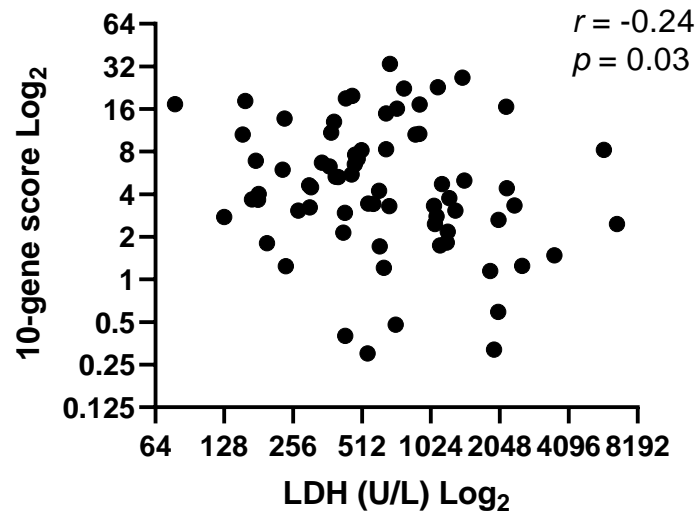

ALL patients

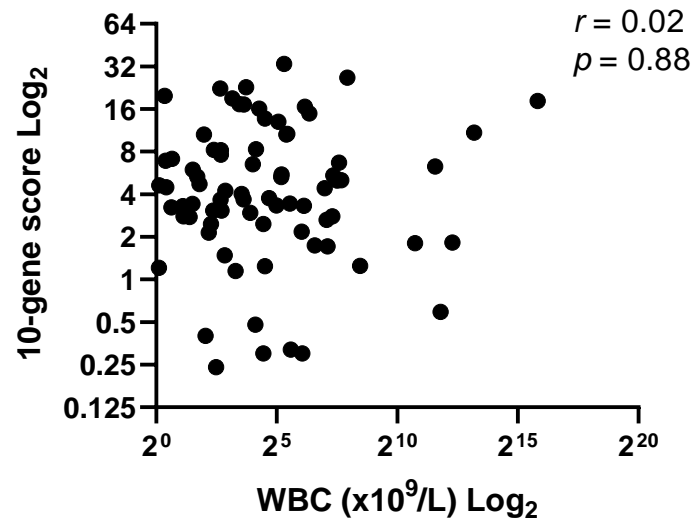

Supplement: Supplementary file 3 — Supporting Information [file JHA2-5-1366-s007.pdf]
