# Supplementary material for: Clinical significance of Philadelphia‐like‐related genes in a resource‐constrained setting of adult B‐acute lymphoblastic leukemia patients
Source: EJHaem. 2024 Oct 7;5(6):1366–9. doi: 10.1002/jha2.1030 (PMC11647732; doi:10.1002/jha2.1030)
Supplement: Supplementary file 4 — Supporting Information [file JHA2-5-1366-s004.docx]

| **Supplementary Table 1**. Association of 10-genes score with clinical and molecular factors in whole B-adult acute lymphoblastic leukemia (B-ALL) cohort. | | | | |
| --- | --- | --- | --- | --- |
| **Factors** | **Whole cohort** | **Low 10-genes score** | **High 10-genes score** | ***p*^1^** |
| B-ALL patients, n | 83 | 24 | 59 |  |
| Age [years; median (minimum-maximum)] | 41 (18 – 87) | 28 (18 – 75) | 47 (18 – 87) | 0.13 |
| Gender, n  Male  Female | 43  40 | 12  12 | 31  28 | 1.00 |
| BM blasts [%; median (minimum-maximum)] | 90 (4 – 100) | 94 (30 – 99) | 89 (4 – 100) | 0.051 |
| PB blasts [%; median (minimum-maximum)] | 54 (0 – 97) | 53 (0 – 97) | 54 (0 -96) | 0.88 |
| BCR::ABL1, n  Positive  Negative | 33  50 | 4  20 | 29  30 | **0.006** |
| CRLF2 rearrangement, n  Positive  Negative  ...N/A | 12  13  58 | 6  6  12 | 6  7  46 | 1.00 |
| Karyotype, n^2^  Good  Intermediate  Poor  ...N/A | 0  30  28  25 | 0  9  7  8 | 0  21  21  17 | 0.77 |
| MRD, n  Positive  Negative  N/A | 15  19  49 | 6  10  8 | 9  9  41 | 0.51 |
| WBC [10^9^/L; median (minimum-maximum)] | 17.4 (1.1 – 57690) | 23.5 (1.1 – 5010) | 14.1 (1.1 – 57690) | 0.23 |
| Hemoglobin [g/dL; median (minimum-maximum)] | 7.6 (4 – 14) | 7.6 (4 – 12.7) | 7.6 (4.8 – 14) | 0.63 |
| Granulocytes [10^9^/L; median (minimum-maximum)] | 1.5 (0 – 1450) | 2.2 (1.1 – 550) | 1.4 (0 – 1450) | 0.29 |
| Platelets [10^9^/L; median (minimum-maximum)] | 32 (3 – 325) | 26 (8 – 325) | 33 (3 – 311) | 0.69 |
| LDH [U/L; median (minimum-maximum)] | 624 (78 – 6653) | 946 (197 – 3545) | 509 (78 – 6653) | **0.02** |

Abbreviations: ALL, acute lymphoblastic leukemia; MRD, measurable residual disease; WBC, white blood cells; LDH, lactic dehydrogenase.

^1^ For statistical analyzes, Mann–Whitney test was used for measured factors, and Fisher's exact test or Chi-squared test was used for categorical factors.

^2^ Cytogenetic risk was stratified according to Moorman (Blood Rev. 2012;26(3):123-35).
