# Supplementary material for: Clinical significance of Philadelphia‐like‐related genes in a resource‐constrained setting of adult B‐acute lymphoblastic leukemia patients
Source: EJHaem. 2024 Oct 7;5(6):1366–9. doi: 10.1002/jha2.1030 (PMC11647732; doi:10.1002/jha2.1030)
Supplement: Supplementary file 5 — Supporting Information [file JHA2-5-1366-s006.docx]

| **Supplementary Table 2.** Primer sequences and concentrations. | | |
| --- | --- | --- |
| **Gene** | **Sequence** | **Concentration** |
| *IFITM2* | FW: GAAGTCTAGGGACAGGAAGATGGTT  RV: AATCAGGGCCCAGATGTTCA | 300 nM |
| *IFITM1* | FW: CCTCTTCTTGAACTGGTGCTGTCT  RV: CGTCGCCAACCATCTTCCT | 300 nM |
| *CD99* | FW: TTGACTTAGGAGATGCTGTTGTTGAT  RV: TTTGGATTTGGCATCGGTTT | 300 nM |
| *CD97* | FW: ACCTACATTTCCCCTTCGAACA  RV: GCTCTGACCCATAGTGACGTTCTT | 300 nM |
| *IGJ* | FW: CATTGAGAACCAGATTTGTGTACCAT  RV: TCTGATTATCCAGCTCCACTTCTGTA | 300 nM |
| *NUDT4* | FW: AAATATTAGAAGATTGGGAAGATTCTGTTAA  RV: TGGAGAACTTTGATAGCATCTTCTACTTT | 300 nM |
| *SEMA6A* | FW: AGCCGTGGATTACGGAGATTATAT  RV: AAAACTACCTTTCCCATGGTGTTATACT | 300 nM |
| *SOCS2* | FW: ATATGTGTCAAATCCAAGCTTAAACAAT  RV: CTTATCCTTGCACATCTGAACATAGTAGT | 300 nM |
| *TP53INP1* | FW: GCTGCTCATACAACTTTTCTGGAA  RV: AGGCTGTCTTTCACTGTGTTCTTTTA | 300 nM |
| *CRLF2* | FW: CCAGACCCGAAATCCATCTTC  RV: CGTTCTGGGTGTCTGTGATCC | 300 nM |
| *GAPDH* | FW: CCACCCATGGCAAATTCC  RV: GATGGGATTTCCATTGATGACA | 300 nM |
| *ATCB* | FW: AGGCCAACCGCGAGAAG  RV: ACAGCCTGGATAGCAACGTACA | 150 nM |
