# Supplementary material for: Clinical significance of Philadelphia‐like‐related genes in a resource‐constrained setting of adult B‐acute lymphoblastic leukemia patients
Source: EJHaem. 2024 Oct 7;5(6):1366–9. doi: 10.1002/jha2.1030 (PMC11647732; doi:10.1002/jha2.1030)
Supplement: Supplementary file 6 — Supporting Information [file JHA2-5-1366-s008.docx]

| **Supplementary Table 3**. Association of 10-genes score with clinical and molecular factors in Philadelphia negative B-adult acute lymphoblastic leukemia (Ph^-^ B-ALL) cohort. | | | | |
| --- | --- | --- | --- | --- |
| **Factors** | **Ph^-^ B-ALL cohort** | **Low 10-genes score** | **High 10-genes score** | ***p*^1^** |
| Ph^-^ B-ALL patients, n | 50 | 20 | 30 |  |
| Age [years; median (minimum-maximum)] | 35 (18 – 87) | 28 (18 – 75) | 43 (18 – 87) | 0.22 |
| Gender, n  Male  Female | 25  25 | 10  10 | 15  15 | 1.00 |
| BM blasts [%; median (minimum-maximum)] | 90 (4 – 100) | 95 (61 – 99) | 88 (4 – 100) | **0.01** |
| PB blasts [%; median (minimum-maximum)] | 55 (0 – 97) | 63 (0 – 97) | 52 (0 – 97) | 0.42 |
| CRLF2 rearrangement, n  Positive  Negative  ...N/A | 12  10  28 | 6  5  9 | 6  5  19 | 1.00 |
| Karyotype, n^2^  Good  Intermediate  Poor  ...N/A | 0  27  5  19 | 0  8  4  8 | 0  18  1  11 | 0.06 |
| MRD, n  Positive  Negative  N/A | 13  19  18 | 6  10  4 | 7  9  14 | 1.00 |
| WBC [10^9^/L; median (minimum-maximum)] | 15 (1.1 – 3550) | 23.5 (1.1 – 3550) | 7.3 (1.3 – 3040) | 0.054 |
| Hemoglobin [g/dL; median (minimum-maximum)] | 7.6 (4 – 14) | 7.6 (4 – 10.2) | 7.5 (4.8 – 14) | 0.96 |
| Granulocytes [10^9^/L; median (minimum-maximum)] | 1.2 (0 – 1450) | 1.9 (0.1 – 520) | 0.8 (0 – 1450) | 0.24 |
| Platelets [10^9^/L; median (minimum-maximum)] | 37 (3 – 325) | 34 (8 – 325) | 52 (3 – 311) | 0.28 |
| LDH [U/L; median (minimum-maximum)] | 610 (128 – 6653) | 812 (197 – 3545) | 476 (128 – 6653) | **0.02** |

Abbreviations: ALL, acute lymphoblastic leukemia; MRD, measurable residual disease; WBC, white blood cells; LDH, lactic dehydrogenase.

^1^ For statistical analyzes, Mann–Whitney test was used for measured factors, and Fisher's exact test or Chi-squared test was used for categorical factors.

^2^ Cytogenetic risk was stratified according to Moorman (Blood Rev. 2012;26(3):123-35).
