# Supplementary material for: Clinical significance of Philadelphia‐like‐related genes in a resource‐constrained setting of adult B‐acute lymphoblastic leukemia patients
Source: EJHaem. 2024 Oct 7;5(6):1366–9. doi: 10.1002/jha2.1030 (PMC11647732; doi:10.1002/jha2.1030)
Supplement: Supplementary file 7 — Supporting Information [file JHA2-5-1366-s003.docx]

| **Supplementary Table 4.** Cox regression analysis for overall survival of B-ALL patients (n=83) according to clinical and laboratorial characteristics and 10-genes score. | | | | | | |
| --- | --- | --- | --- | --- | --- | --- |
| **Factors** | **Univariate analysis** | | | **Multivariate analysis** | | |
|  | **H.R.^1^** | **(95% C.I.)** | ***p*** | **H.R.** | **(95% C.I.)** | ***p*** |
| Gender  Male *vs.* female |  |  |  |  |  |  |
|  | 1.34 | 0.74-2.44 | 0.329 | 1.39 | 0.71-2.74 | 0.333 |
| Age at diagnosis (years)^2^ | 1.02 | 1.00-1.04 | **0.004** | 1.02 | 1.00-1.04 | **0.015** |
| White blood cell count (10^9^/L)^2^ | 1.00 | 0.99-1.00 | 0.673 | 1.00 | 0.99-1.00 | 0.817 |
| Hemoglobin (g/dL)^2^ | 1.09 | 0.95-1.26 | 0.192 | 1.05 | 0.86-1.29 | 0.637 |
| Platelets (10^9^/L)^2^ | 0.99 | 0.99-1.00 | 0.678 | 0.99 | 0.99-1.00 | 0.181 |
| Granulocytes (10^9^/L)^2^ | 1.00 | 0.99-1.00 | 0.380 | 1.00 | 0.99-1.00 | 0.111 |
| LDH (U/L)^2^ | 1.00 | 0.99-1.00 | 0.291 | 1.00 | 0.99-1.00 | 0.158 |
| Cytogenetic risk^3^  Poor *vs.* intermediate |  |  |  |  |  |  |
|  | 0.85 | 0.43-1.69 | 0.646 | 0.43 | 0.16-1.15 | 0.093 |
| Unknown *vs*. intermediate | 1.11 | 0.54-2.29 | 0.777 | 0.88 | 0.41-1.89 | 0.738 |
| *BCR::ABL1*  Positive *vs.* negative |  |  |  |  |  |  |
|  | 1.18 | 0.65-2.11 | 0.579 | 0.95 | 0.39-2.27 | 0.909 |
| 10-genes score  High *vs.* low |  |  |  |  |  |  |
|  | 2.28 | 1.10-4.78 | **0.026** | 2.66 | 1.15-6.16 | **0.023** |

Abbreviations: H.R., hazard ratio; C.I., confidence interval; LDH, lactic dehydrogenase; MRD, measurable residual disease.

Significant statistical differences are highlighted in bold.

^1^ Hazard ratios (HR)> 1 indicates that the increase in values for continuous variable or the first factor for categorical variable has a worse outcome.

^2^ Factors were analyzed as continuous variables.

^3^ Cytogenetic risk was stratified according to Moorman (Blood Rev. 2012;26(3):123-35).
