# Supplementary material for: Clinical significance of Philadelphia‐like‐related genes in a resource‐constrained setting of adult B‐acute lymphoblastic leukemia patients
Source: EJHaem. 2024 Oct 7;5(6):1366–9. doi: 10.1002/jha2.1030 (PMC11647732; doi:10.1002/jha2.1030)
Supplement: Supplementary file 8 — Supporting Information [file JHA2-5-1366-s005.docx]

| **Supplementary Table 5.** Cox regression analysis for overall survival of Ph^-^ B-ALL patients (n=50) according to clinical and laboratorial characteristics and 10-genes score. | | | | | | |
| --- | --- | --- | --- | --- | --- | --- |
| **Factors** | **Univariate analysis** | | | **Multivariate analysis** | | |
|  | **H.R.^1^** | **(95% C.I.)** | ***p*** | **H.R.** | **(95% C.I.)** | ***p*** |
| Gender  Male *vs.* female |  |  |  |  |  |  |
|  | 2.13 | 0.92-4.91 | 0.08 | 3.75 | 1.36-10.39 | 0.011 |
| Age at diagnosis (years)^2^ | 1.03 | 1.01-1.05 | **0.004** | 1.04 | 1.02-1.08 | **0.002** |
| White blood cell count (10^9^/L)^2^ | 1.00 | 1.00-1.001 | **0.027** | 1.00 | 1.00-1.00 | **0.019** |
| Hemoglobin (g/dL)^2^ | 1.07 | 0.86-1.33 | 0.567 | 0.96 | 0.66-1.42 | 0.864 |
| Platelets (10^9^/L)^2^ | 0.99 | 0.98-1.00 | 0.193 | 0.98 | 0.97-0.99 | **0.011** |
| Granulocytes (10^9^/L)^2^ | 1.00 | 0.99-1.00 | 0.152 | 1.00 | 0.99-1.00 | 0.693 |
| LDH (U/L)^2^ | 1.00 | 0.99-1.00 | 0.642 | 1.00 | 0.99-1.00 | 0.053 |
| Cytogenetic risk^3^  Poor *vs.* intermediate |  |  |  |  |  |  |
|  | 0.72 | 0.16-3.19 | 0.669 | 0.75 | 0.14-3.92 | 0.732 |
| Unknown *vs*. intermediate | 0.91 | 0.39-2.11 | 0.823 | 0.48 | 0.16-1.42 | 0.185 |
| 10-genes score  High *vs.* low |  |  |  |  |  |  |
|  | 2.39 | 0.99-5.78 | 0.051 | 5.72 | 1.62-20.14 | **0.007** |

Abbreviations: H.R., hazard ratio; C.I., confidence interval; LDH, lactic dehydrogenase; MRD, measurable residual disease.

Significant statistical differences are highlighted in bold.

^1^ Hazard ratios (HR)> 1 indicates that the increase in values for continuous variable or the first factor for categorical variable has a worse outcome.

^2^ Factors were analyzed as continuous variables.

^3^ Cytogenetic risk was stratified according to Moorman (Blood Rev. 2012;26(3):123-35).
